# Disease Ecology Camp: A Model for Engaging Undergraduates in Outreach with Underserved Youth

**Published:** 2025-02-12

**Authors:** Hilarie Davis, Bradford Davey, Jamie Cornish, Nora Smith, Tugba Boz, Rebekah Hammack

**Affiliations:** 1 Technology for Learning Consortium, Inc., Stuart, FL;; 2 Academic Technology and Outreach, Montana State University, Bozeman, MT;; 3 College of Education, Purdue University, West Lafayette, IN

**Keywords:** Disease Ecology, Undergraduate Outreach, Underserved, Middle School Youth, Place-Based, Indigenous Science

## Abstract

Disease ecology classes were conducted at two residential middle school summer camps held at a university—a camp for local youth who paid tuition and a free camp for youth from statewide underserved communities including Native, low-income, and rural. Disease ecology is defined as the ecological study of host-pathogen interactions within the context of their environment and evolution. The classes were created by a grant-funded program for disease ecology designed to increase middle school youths’ interest, confidence, and identity in STEM, and their understanding of disease ecology. Undergraduate researchers were recruited to develop and teach lessons in disease ecology. This paper outlines the project’s elements, effects on undergraduate students and middle school youth, and lessons learned. This study has three key findings: 1) Undergraduate participants increased their confidence and interest in science outreach and their understanding of disease ecology, 2) STEM identity increased in both underserved middle school youth and a comparison youth group, 3) Both underserved and comparison group middle school youth increased their understanding of disease ecology, Native culture, traditional knowledge about food, how to be healthy where they live, and how science can help their communities.

## INTRODUCTION

Our project involved training undergraduates to develop culturally relevant, place-based science lessons for middle school youth on disease ecology – an emerging field that focuses on the relationship of hosts and pathogens in the environment over time (Oppong and Huddleston, 2014). Place-based science education situates the teaching and learning of science in the learners’ physical context – where they are (Smith, 2002, Sobel, 2004). It is widely recognized as a successful approach for engaging diverse learners and attracting underserved audiences into sciences, as evidenced by various studies (Lim and Calabrese Barton, 2006). It has proven particularly helpful with at-risk populations (Avery, 2013; McInerny, et al., 2011), engaging a diverse group of youth in relevant activities (Emekauwa, 2004), and strengthening youths’ connections and sense of belonging in rural communities (Bouchard, 2022; Sobel, 2004). This paper reports on a health science project designed to explore how such an approach can be utilized to engage diverse and underrepresented students in STEM fields.

In this initiative, undergraduate researchers had the opportunity to enhance their communication and outreach skills under the guidance of university STEM education faculty. The importance of these skills is increasingly recognized in practice and in the literature, as scholars highlight the need for scientists to communicate their work and engage public audiences (Haywood and Besley, 2014; Lopes, et al., 2018; Stony Brook University, 2009). Results from projects and studies show that through engaging in outreach, undergraduates can deliver new content to middle school youth while also developing their skills, confidence, and interest in outreach (Varner, 2014; Volbrecht, et al., 2019; Yawson, et al., 2016). Such outreach events serve as opportunities both to engage youth in STEM (Freeman, et al., 2014; Prince, 2004) and to improve undergraduates’ skills and confidence (Nelson, et al., 2017).

The core of the project involved a collaborative effort in which the project team, consisting of university STEM education faculty and staff and an external evaluator, worked closely with undergraduates to identify and/or create disease ecology activities. These activities were designed to be engaging and effective by incorporating elements of place-based investigations, teamwork, cultural relevance, and integrated content in disease ecology. The process began in September 2022 with the recruitment and training of undergraduate students who then met bi-weekly to work on their activities. During the academic year, students had opportunities to test the activities at events like university STEAM day. The activities focused on microbes, water quality, nutrition, and local diseases (e.g., rocky mountain spotted fever, blue tongue, giardia) designed to be used in two middle school science camps held at the university – an underserved camp for qualifying students, and the second, a comparison camp, for students who paid tuition. Five of the undergraduates, including two Indigenous students, became instructors for the camps and facilitated a daily disease ecology class of 90 minutes, over five days, totaling 7.5 hours of contact time. The project objectives for the undergraduates were to (1) increase their interest, skills, and confidence with regard to science outreach and communication, and (2) improve their understanding of disease ecology and their confidence in teaching others about the topic.

The project objectives for middle school youth were to (1) improve their STEM identity and (2) develop an understanding of disease ecology and how it can help them and their communities stay healthy. The evaluator conducted an analysis of the impact of the activities on participating youth to see if the model was effective with underserved youth because in the state where this project was conducted, only about 36% of youth are proficient in science (Montana State Report Card, 2022). Youth in the state attend a diverse range of school districts and educational contexts, including 12 federally recognized tribal nations (11% of school-age youth). Because of the sizable population of Indigenous youth in the state, the project was designed to respect Indigenous knowledge systems of place, backgrounds, and identities (Bruchac, 2016; Peltier, 2021; Pineda, et al., 2020; Smith, 2021; Windchief and San Pedro, 2019).

In this paper, we report on the effect of the project on participating undergraduates and middle school youth. Specifically, our study questions are: Q1: Does learning about and doing disease ecology outreach affect undergraduates’ confidence, skills, and interest in science communications and their understanding of disease ecology? Q2: Does attending disease ecology camp affect middle school youths’ STEM Identity, particularly underserved youth? Q3: Does attending disease ecology camp affect middle school youths’ understanding of disease ecology (particularly underserved youth), including Native culture and traditional knowledge, how to be healthy, and how science can help?

## PROGRAM DESCRIPTION

**Figure 1.**
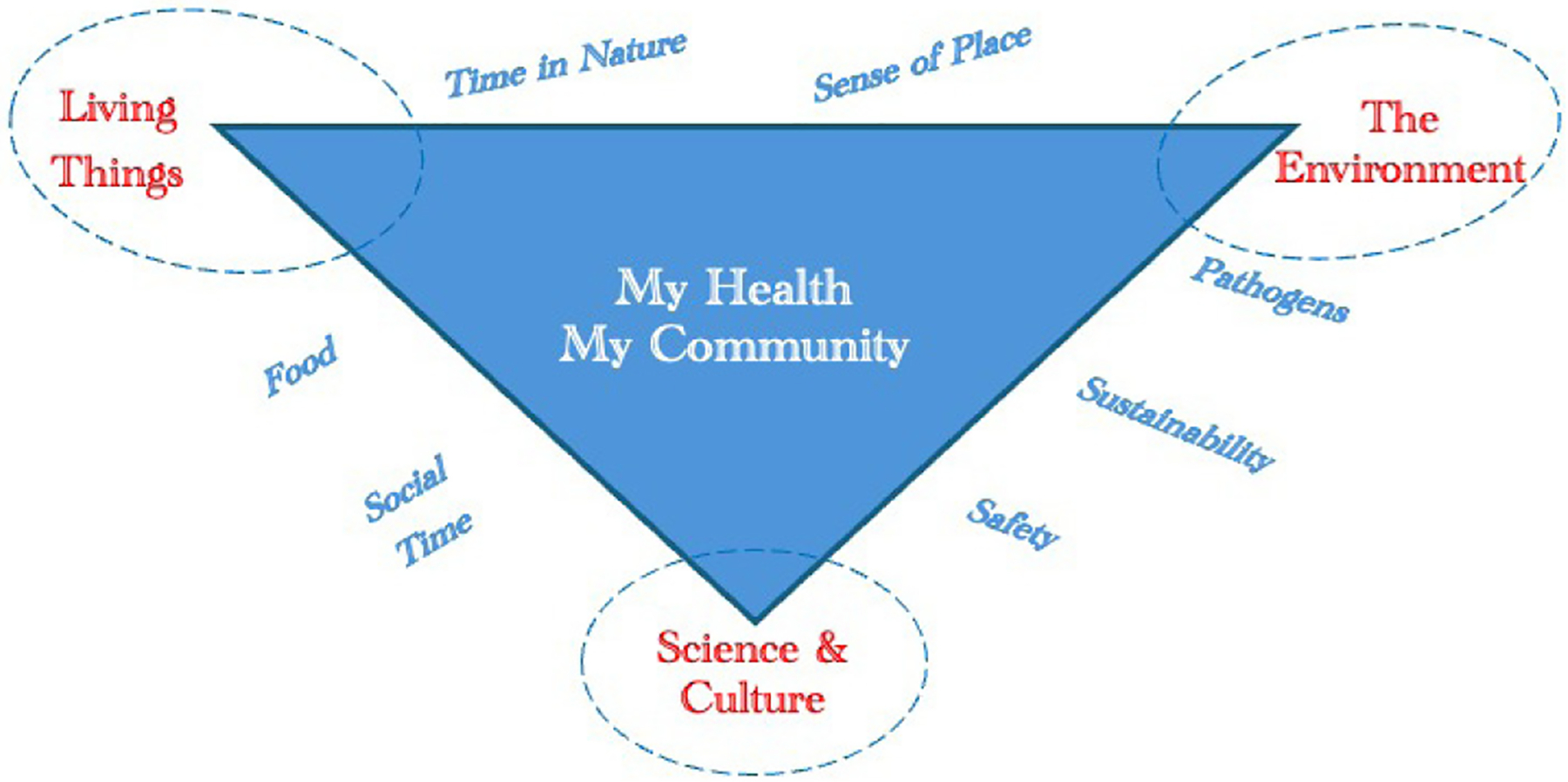
Visual model of disease ecology developed from a literature review.

Undergraduates were recruited in September 2022 and attended a three-hour training about the program goals, science outreach, human subjects research, the Next Generation Science Standards (NGSS), and integrating Indigenous knowledge. This initial training was offered on three different occasions by the project’s STEM educators to accommodate the schedules of the students at the four universities. The undergraduates who joined the project identified areas of interest and formed teams to develop activities. They were provided a visual model of the key concepts in disease ecology (see Figure 1) derived from the literature.

The undergraduate teams met with the project STEM educators regularly to learn more about disease ecology and the resources to teach it. In May 2023, they presented their final lessons to the project team. A subset of activities were chosen for the camp. The schedule for campers was three 90-minute science classes a day for five days. The disease ecology activities for the week were: Day 1—an introduction to disease ecology and microbes, Day 2—isolating and identifying microbes on or around you, Day 3—water systems and sources of pollution, Day 4—nutrition and Native science, and Day 5—microplastics, water filtration, and algal blooms. The pathogens studied were local so students knew people or animals who had been affected (e.g., Rocky Mountain spotted fever, giardia, covid, blue tongue disease. (See Table 1).

The three main concepts covered in the camp were revisited every day as youth added to their concept maps and took on the three big ideas:

1. Living things interact with each other in the environment in good and bad ways.
2. How you interact with your environment can affect your health.
3. Scientists work to improve our health—and you can help too.

At the beginning and end of the year, the undergraduates created concept maps to show their understanding of disease ecology and completed an evaluator-developed survey on the effects of their experience. Twenty-five undergraduates signed up for the project; twelve completed lessons, and five students were available to teach in the summer camps.

## METHODS

### Participants.

**Figure 2.**
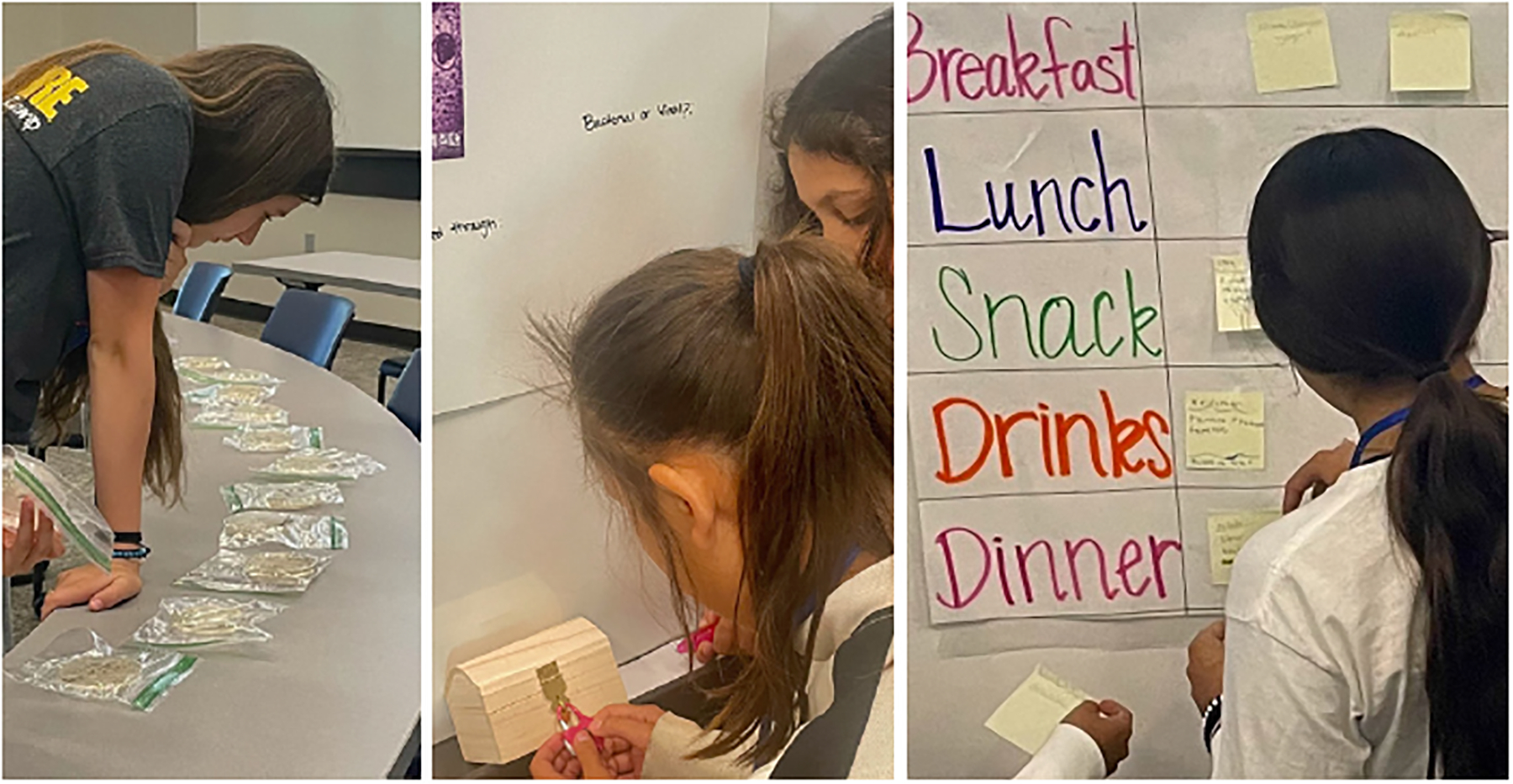
Photos of Youth Doing Activities. (*v1*)

The undergraduate students (N=25) were recruited from those doing health sciences research as part of the NIH INBRE program who were interested in learning about disease ecology and outreach with middle school youth (Figure 2). Middle schoolers (N=72) were recruited for the two one-week camps. Additional details about the two groups are given below.

#### Undergraduates.

The undergraduates were recruited from two public universities and two tribal colleges to identify or develop lessons in disease ecology for middle school youth. The undergraduates were recruited from the IDeA Networks of Biomedical Research Excellence (INBRE) program. Funded by NIH, INBRE funds statewide networks of higher education and research institutions to build biomedical research capacity through support for faculty research and mentoring, student participation in research, and research infrastructure enhancement at network institutions. There were 25 undergraduates who initially signed up for the project (8 males, and 17 females). The undergraduates were sophomores, juniors and seniors. University students’ majors included biology, microbiology, civil engineering, environmental science, neuroscience, and exercise physiology. Tribal college student majors were in Native studies, liberal arts, health sciences, business administration, and environmental studies.

#### Middle School Campers.

The project partnered with two residential middle school camps held at the university—one, a free camp for underserved youth, (N=32) and the second, comparison group camp ($789 tuition) for youth from the region (N=40). The demographics of the groups were similar for gender, with more Native Americans in the underserved group and more Asian Americans in the comparison group. Students could choose more than one ethnicity category: Underserved group 2% Asian American, 7% African American, 28% Native American, 7% Hispanic, 47% White, 52% male, and 43% female; Comparison group 25% Asian American, 15% African American, 9% Native American, 8% Hispanic, 26% White, 62% male, and 23% female. Youth in the underserved camp met one or more of the following criteria to qualify: in the free/reduced lunch program, had parents who did not attend college, attended a school with low college attendance and low rates of math and English proficiency, lived in rural areas, or were from a minority group.

### Data Collection and Analysis.

The measures used to investigate the study questions are described below.

Q1: Does learning about and doing disease ecology outreach affect undergraduates’ confidence, skills, and interest in science communications and their understanding of disease ecology?

Data were collected from undergraduates twice during the academic year (1) in September 2022, initial concept maps of disease ecology and (2) in May 2023 at the end of the academic year, a final concept map of disease ecology and an evaluator developed survey.

Concept maps were used to investigate undergraduates’ understanding of disease ecology. A concept map is a graphical tool to activate and elaborate on prior or gained knowledge. Open-ended mapping (beginning with one concept) engages the learner in evaluative thinking, generating ideas, and confronting gaps in knowledge and relationships of concepts (Ruiz-Primo, et al., 2001). They change over time as knowledge grows (Daley and Torre, 2010). These were scored using Novak and Gowin’s system (1984). In their system, scores are given as a measure of understanding for concepts (1 point/concept), links (1 point/valid relationship), number of branches (1 pt for first, 3 points for others), each hierarchy of concepts (5 points each), and crosslinks (10 points each). Two evaluators independently scored each map, then the scores were compared and discrepancies resolved.

At the end of the year the undergraduates rated themselves retrospectively before and after the project (scale=1–10, where 1=none and 10=very high) on interest in science communication and outreach, its use in helping the community, their skills, and their confidence. Undergraduates were also asked to rate the value of the project (worth their time), enjoyment, and likelihood of recommending the project to someone else. They rated the importance of science communication on a scale of 1–4 (1=strongly disagree, 4= strongly agree). In response to open-ended questions, they reflected on what they liked about the project and how it benefitted them.

Q2: Does attending disease ecology camp affect middle school youths’ STEM Identity?

**Figure 3.**
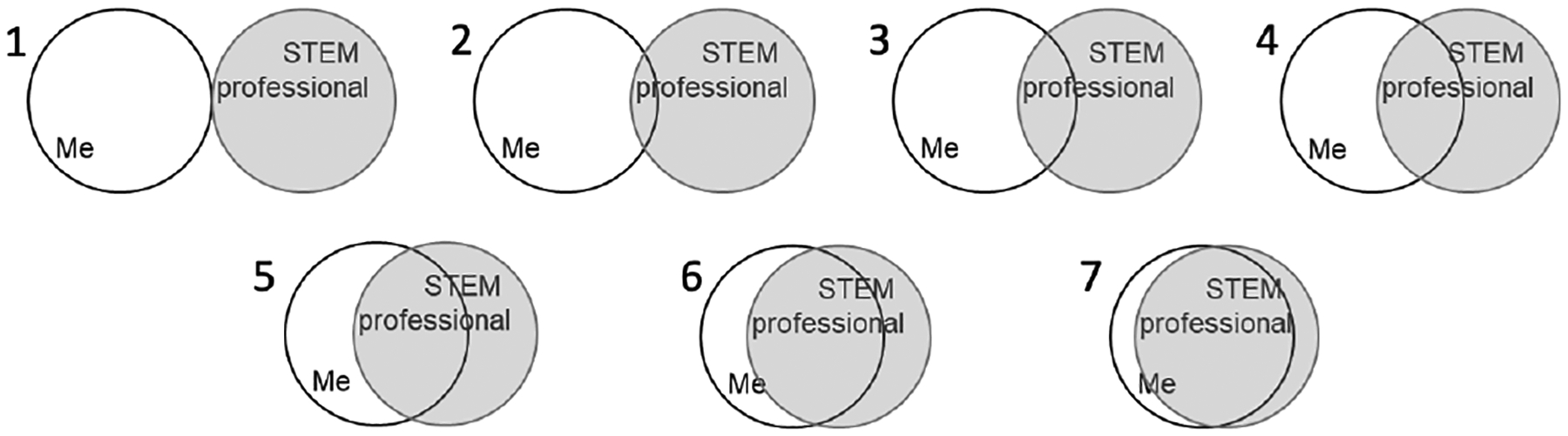
STEM Identity measure.

A validated STEM identity measure (McDonald, et al., 2019) asked youth to rate themselves retrospectively before and after the camp on the overlap between the image of themselves and their image of a STEM professional (1–7, 7=mostly overlapped, (see Figure 3). Pre/post ratings were analyzed using a t-test for matched pairs.

The second part of the end-of-camp survey was a retrospective question developed by the evaluator on the outcome objectives of the disease ecology camp that asked youth to rate themselves before and after the camp (scale=1–10, 1=none at all, 10=very high)) on confidence they can do well in science, understanding of disease ecology, working with others to do an investigation, understanding Native culture, understanding traditional knowledge about food, knowing how to be healthy where they live, and how science can help their community.

Q3: Does attending disease ecology camp affect middle school youths’ understanding of disease ecology, including Native culture and traditional knowledge, how to be healthy, and how science can help?

Concept maps on disease ecology were created by youth on the first day which they added to during the week, resulting in a final version. As with the undergraduate maps, the beginning and final versions were scored using Novak and Gowin’s system (1984) by two evaluators and differences resolved through discussion.

## RESULTS

Results are presented for undergraduates followed by results from youth at the summer camps.

### Results for Undergraduate Students.

Q1: Does learning about and doing disease ecology outreach affect undergraduates’ confidence, skills, and interest in science communications, and their understanding of disease ecology?

On the end-of-the-year survey, undergraduates reported gains on a scale of 1–10 (1=none, 10=very high) in their interest in science communication and outreach (7.0 before, 8.8 after), its use in helping the community (5.3, 8.7), their skills in communication and outreach (4.9, 7.7), and their confidence in communication and outreach (4.5, 7.8) (Table 2).

One open-ended question asked undergraduates to reflect on the unique aspects of the project and its benefits. They reported that the project provided consistent support from the project team, focused on a specific audience (middle school), comprehensive training, a required cultural connection, and a clear and well-defined approach to outreach. They reported that the biggest benefits of this project to them were learning about Indigenous culture, knowing about state-specific diseases, getting to work with other students (hear their perspectives), and working on things they are passionate about (health, food, community). Student teams developed or adapted 11 activities. They also reported that the project helped them communicate what they were thinking by talking about it every week, that they learned how to make a fun activity in which people learn, and it made them understand and value communicating their science even more. For example, a participating undergraduate reported, “It [the project] reinforced my identity as a researcher. This project has shown me that if I articulate my ideas and have confidence, I could have a real impact.” Another participant reflected, “[The project] Made us think about what a middle schooler actually needs and how to make it stick in their brain.”

**Figure 4.**
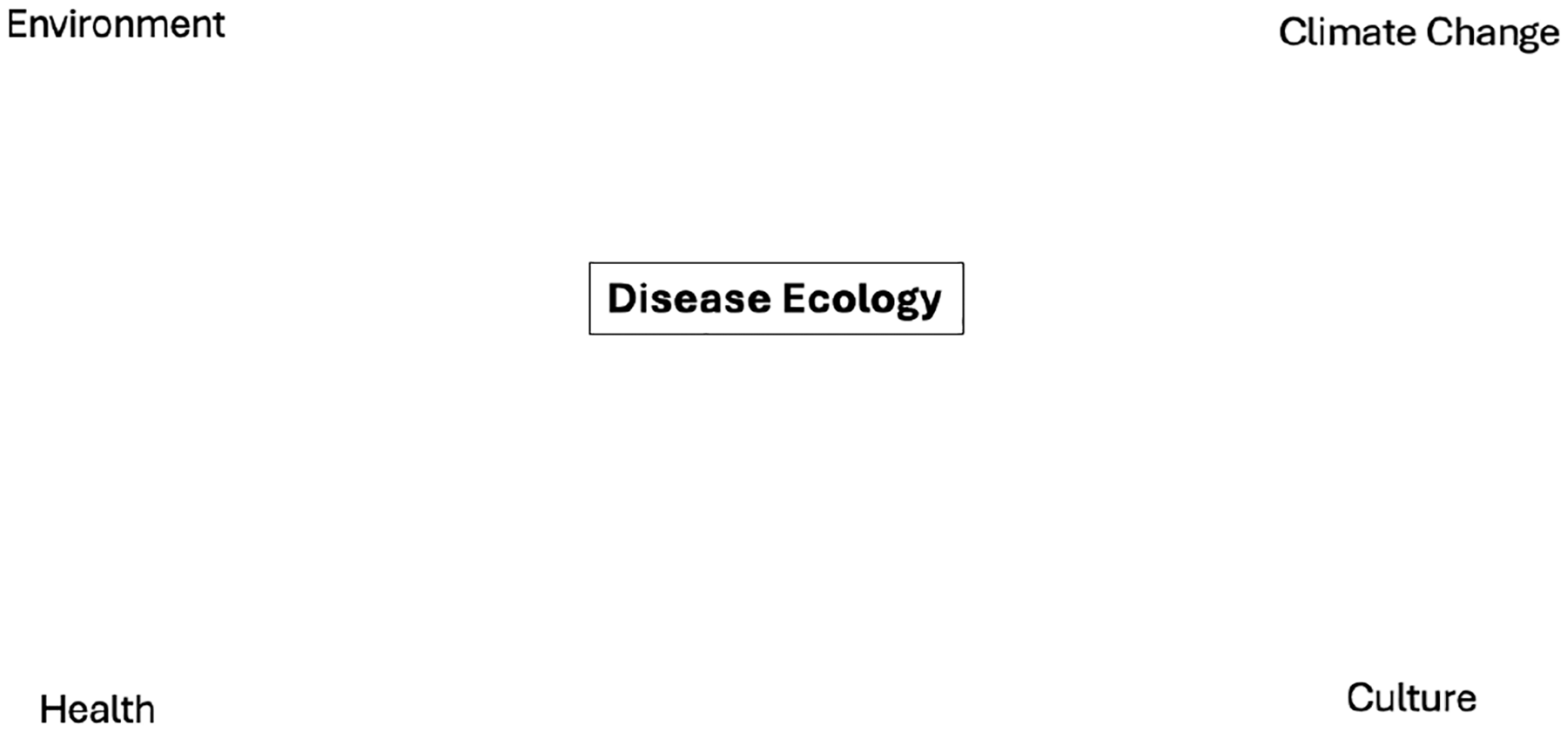
Undergraduate beginning of the year concept map.

**Figure 5.**
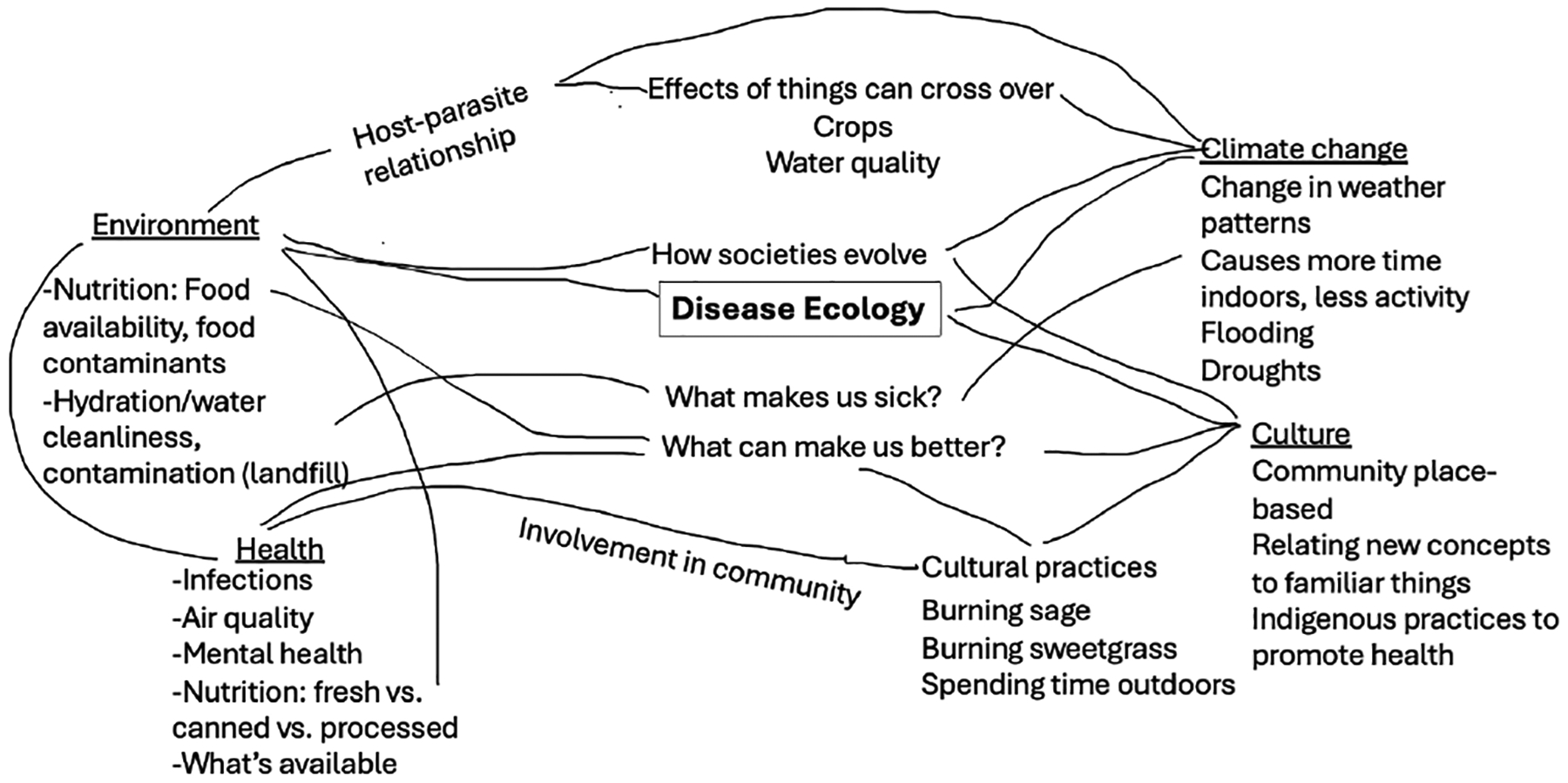
Undergraduate end of the year concept map.

The undergraduates were asked to develop their own disease ecology concept maps before and after the program. Due to the scheduling issues and the logistics of working with undergraduates across four campuses, only about half (12/25) of undergraduates completed concept maps at the end of the year (May 2023). Although not enough were completed to do a statistical analysis, their gains showed positive change. They were scored using Novak and Gowin’s system (1984). See scores in Table 3 and sample pre/post concepts maps in Figures 4 and 5.

### Results for Middle School Youth.

As described in the Program section, the campers in both the underserved and comparison camps participated in the same disease ecology lessons for five days for 90-minute per day. Data were collected to determine if the disease ecology program was equally effective with the underserved and comparison groups and what the effects were on their STEM identities and understanding of disease ecology in the context of their lives and communities.

Q2: Does attending disease ecology camp affect middle school youths’ STEM Identity?

**Figure 6.**
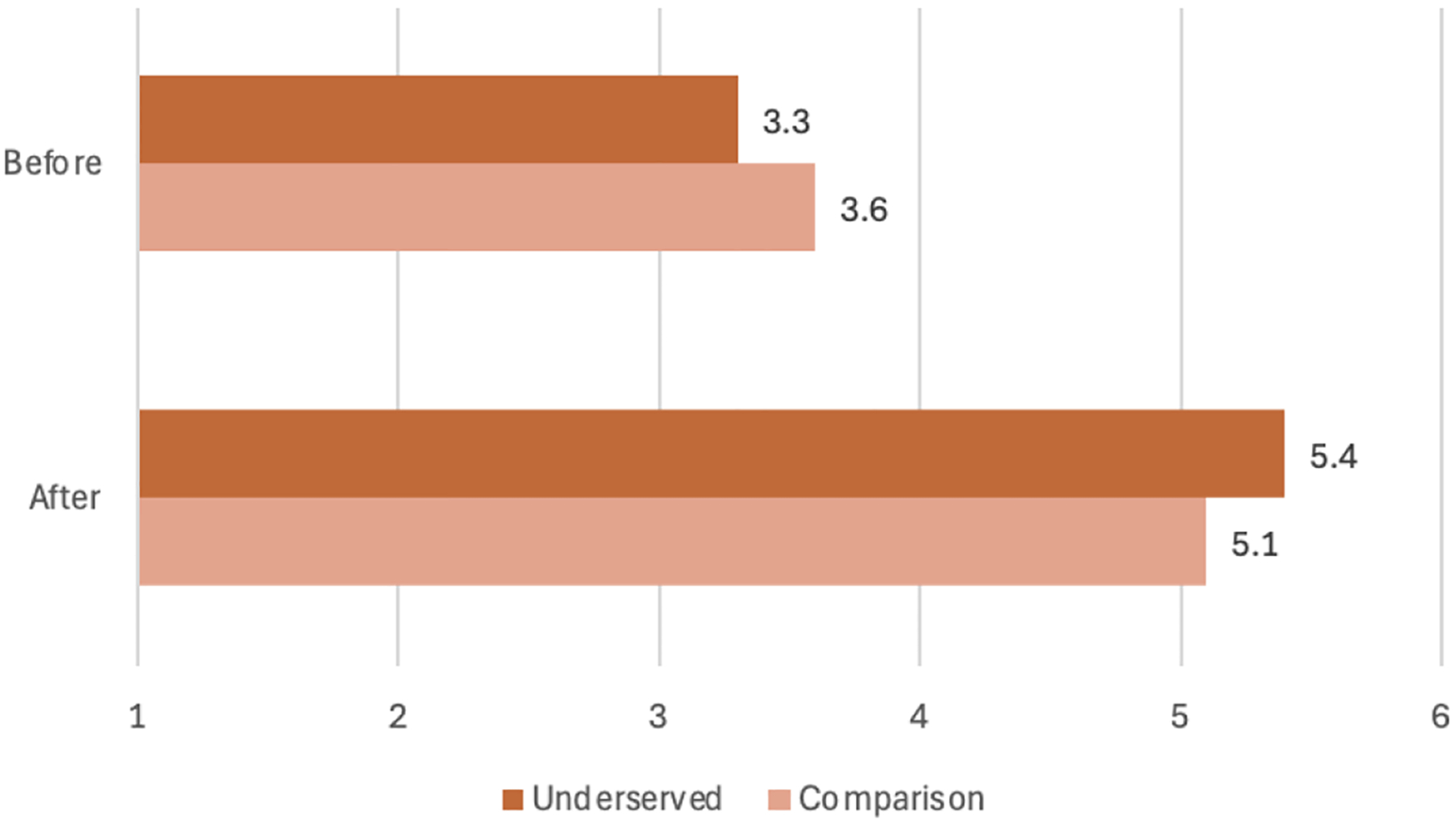
Graph of Youth STEM Identity before and after camp by group.

Youth were asked to indicate how much they identified with a STEM professional before and after the camp on a validated measure with a scale of 1–7 (7=most overlapped) in a retrospective survey. Both groups showed gains on a t-test for matched pairs with the underserved group gaining from 3.3 to 5.4 (p<0.05, Table 4). The comparison group started slightly higher (3.6) while increasing less (gain of 1.5 vs. 2.1 gain) than the underserved group Figure 6).

Q3: Does attending disease ecology camp affect middle school youths’ understanding of disease ecology, including Native culture and traditional knowledge, how to be healthy, and how science can help?

A program specific survey was developed by the evaluator with the project team to measure youths’ before and after understanding of disease ecology, how they can be healthy where they live, how science can be used to help their community, Native culture around health, and traditional food (1–10 scale on pre/post retrospective).

**Figure 7.**
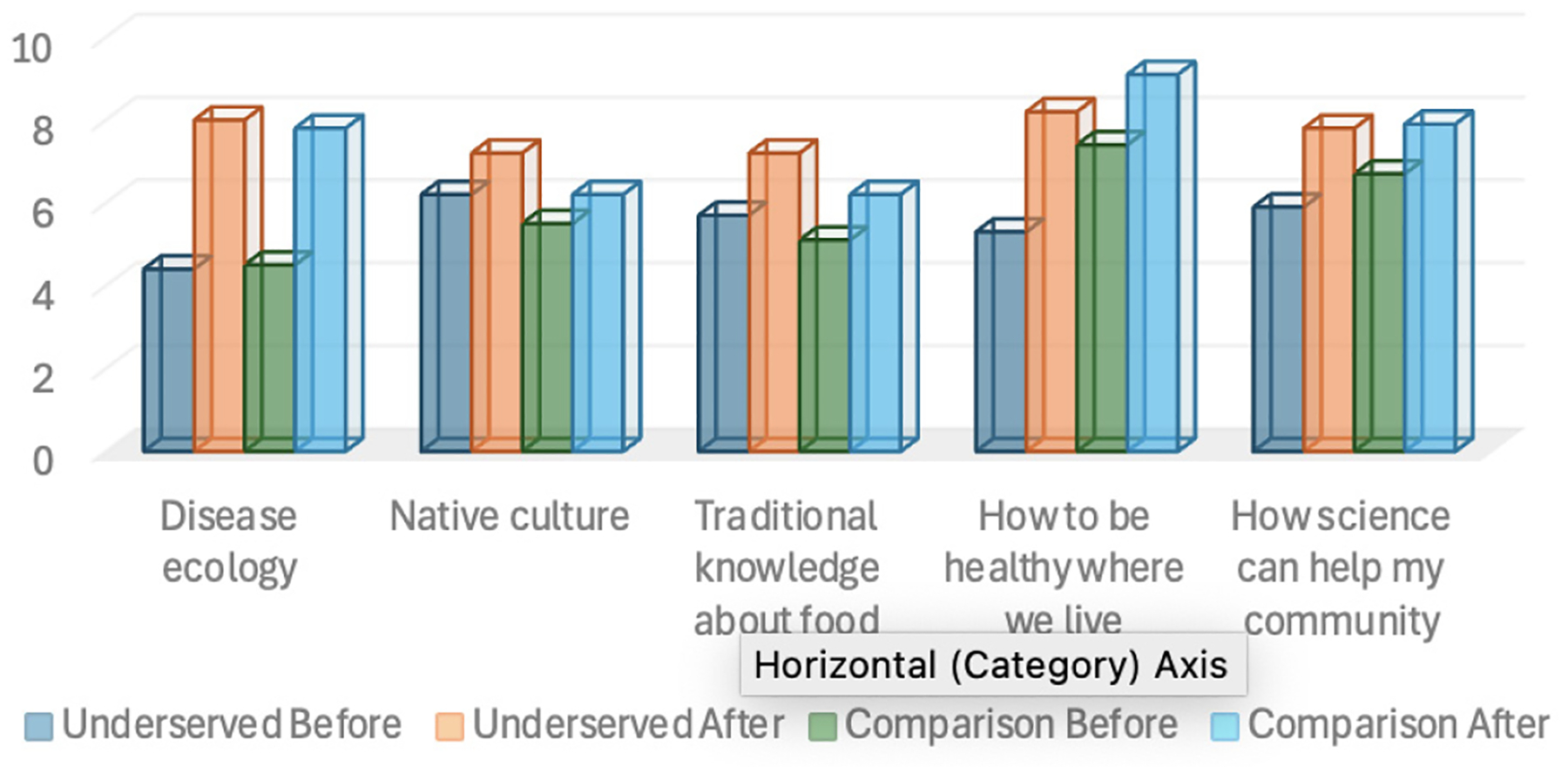
Underserved vs. Comparison Group Effects on Youth Understanding.

Both groups reported significant increases from before to after the camp on all five questions (p<0.05 on a t-test for matched pairs). In a retrospective question at the end of the camp, students were asked to think back to before the camp and rate their understanding on a scale of 1–10, then rate their understanding after the camp. Average ratings on the five items after the camp were similar at 7.7 for the underserved group and 7.4 for the comparison group, while the underserved group had a lower rating than the comparison group before at 5.5 vs. 5.8, resulting in an average higher gain for the underserved group (2.2 vs. 1.6), as shown in Table 5. These data suggest that both youth groups benefitted from the camp with the underserved group making slightly larger gains pre/post (see Figure 7).

Beginning and end of the camp disease ecology concept map scores were compared for the two groups and increases calculated. Two scorers used Novak and Gowin’s system (1984), and resolved discrepancies through discussion. Post map scores for the underserved group (78.4) were much higher than the comparison group (45.3) with nearly twice the gain from pre to post (+68.6 for the underserved group and +36.8 for the comparison group, Table 6).

**Figure 8.**
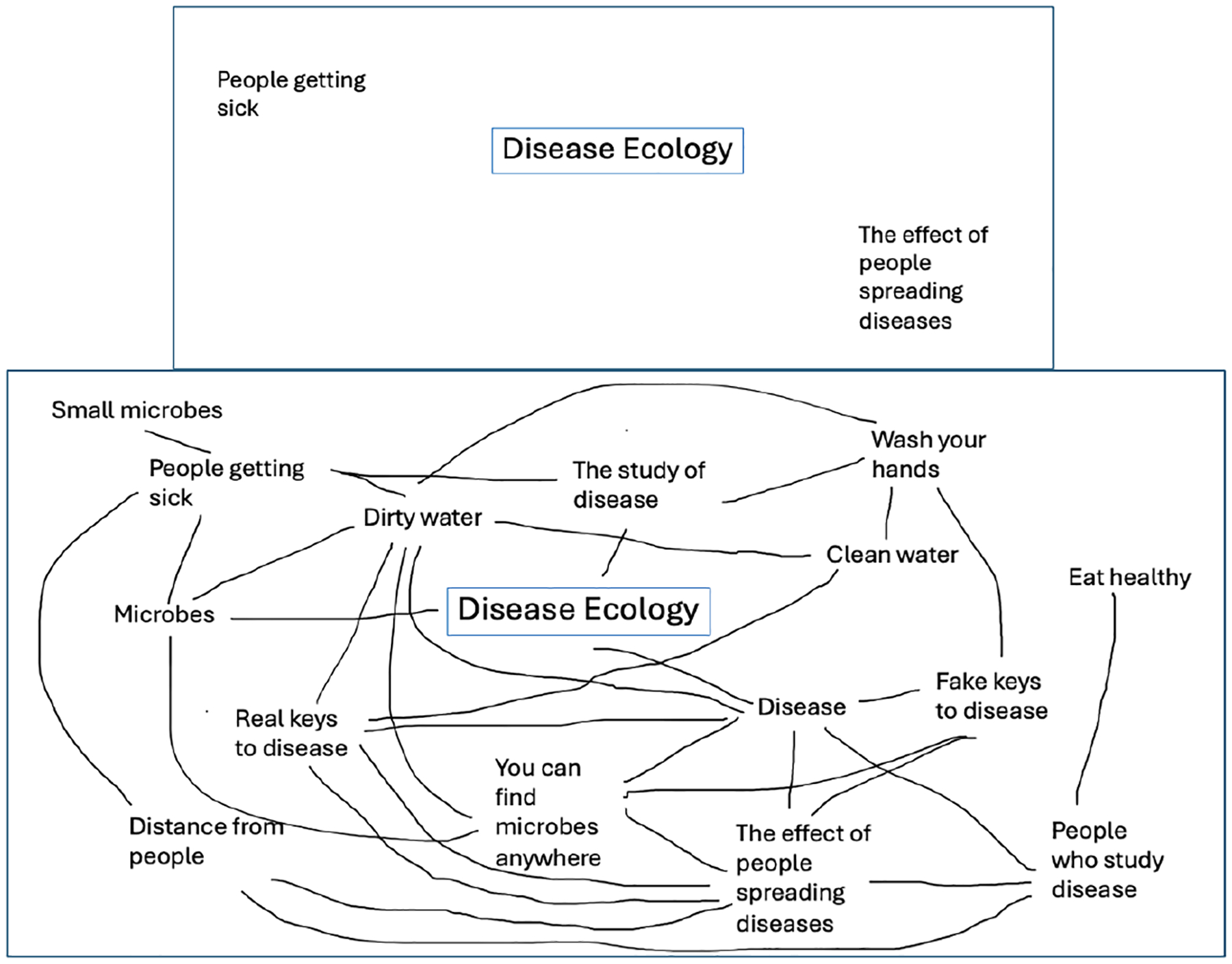
Underserved Youth Group Pre/Post Disease Ecology Concept Map Example.

**Figure 9.**
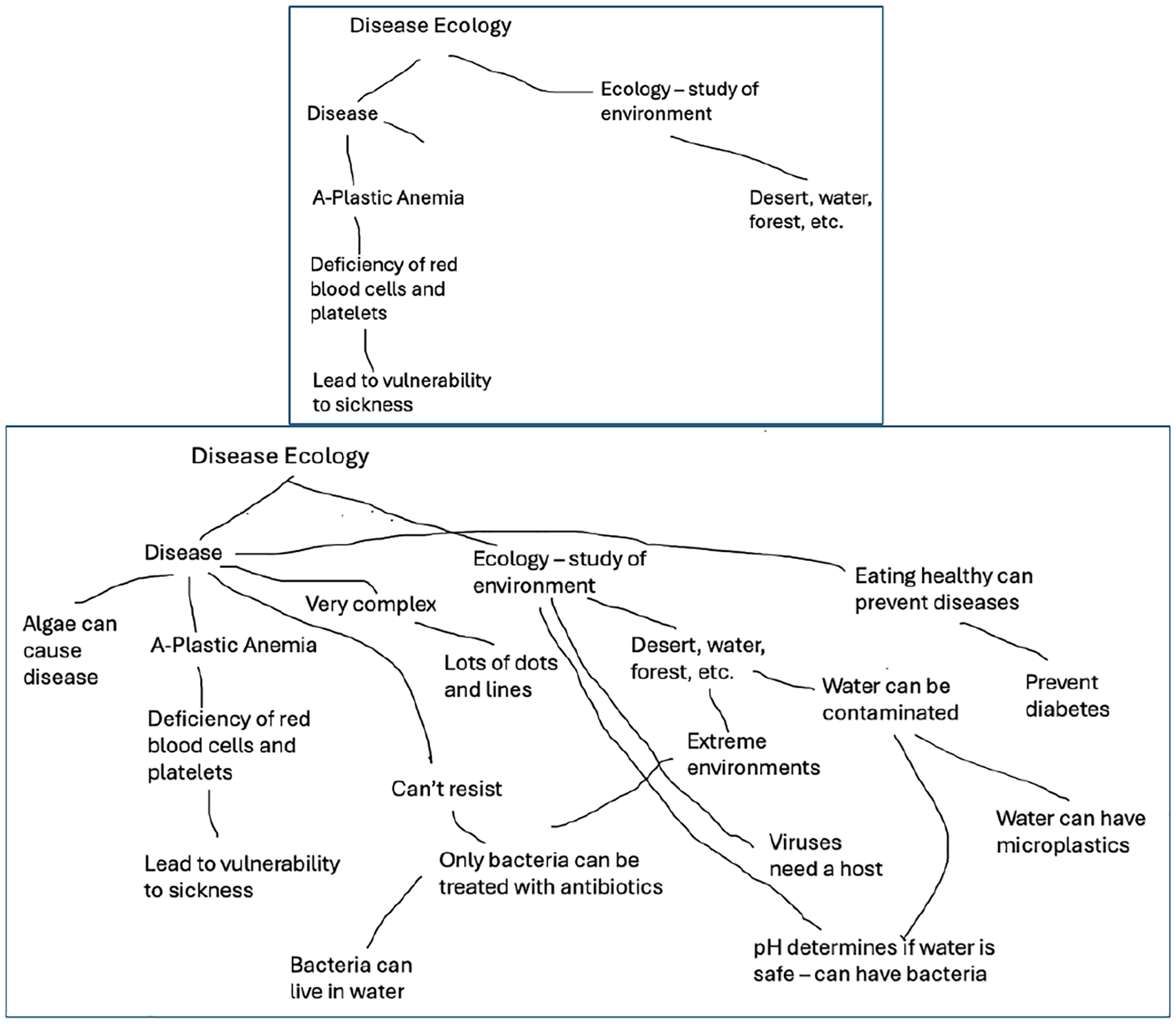
Comparison Youth Group Pre/Post Disease Ecology Concept Map Example.

Below are two pre/post examples of disease ecology maps from youth (Figures 8 and 9).

## DISCUSSION

This project developed and tested a model for engaging undergraduates in outreach with middle school youth in camps using disease ecology with place-based activities. Undergraduates were from public universities and tribal colleges. Campers were from underserved rural and indigenous communities who attended a one-week free camp and a second camp who paid tuition and served as a comparison group. Both camps had the same disease ecology classes.

Undergraduates were recruited to work with science and STEM education faculty at four universities to identify or develop disease ecology activities for middle school youth. They received initial training and ongoing support from STEM education faculty throughout the year. Twenty-five undergraduates worked in teams to develop 11 activities. By their own report on retrospective questions at the end of the program, participating in the program increased their confidence, interest, and valuing of science communication and outreach, and their understanding of disease ecology and how to engage middle school youth in thinking about their own health and the health of their communities. This is consistent with the findings of others (Nelson, et al., 2017; Varner, 2014; Volbrecht, et al, 2019; Yawson, et al., 2016). This model shows how a relevant topic like disease ecology (place-based and culture-connected) can be used to engage underserved youth in STEM.

A subset of the activities developed were used in the two summer STEM camps. Both groups showed significant gains in understanding of disease ecology, Native culture, understanding of Indigenous knowledge, traditional knowledge of food, how to be healthy where they live, and how science can help their community. Both groups reported significant increases from before to after the camp in all five areas. Average ratings on the five items after the camp were similar at 7.7 for the underserved group and 7.4 for the comparison group, but the underserved group had a lower average rating before at 5.5 vs. 5.8 for an underserved group average gain of 2.2 vs. 1.6 for the comparison group. These data suggest that both groups benefitted from the camp with the underserved group perhaps benefitting more.

Youth were asked to indicate how much they identified with a STEM professional before and after the camp on a validated measure (McDonald, et al., 2019) using a scale of 1–7 in a retrospective question. Both groups showed gains in STEM Identity with the underserved group gaining from 3.3 before to 5.4 after, a gain of 1.8. The comparison group started slightly higher and increased less (3.6 to 5.1, +1.5). It is important to note that both groups improved their STEM identity as a result of the camp activities.

As an indicator of understanding disease ecology, youth concept maps were scored and compared. Post map scores for the underserved group (mean score of 78.4) were much higher than the comparison group (mean score of 45.3) with nearly twice the gain from pre to post (+68.6 for underserved and +36.8 for the comparison group). These scores indicate that while both groups knew very little about disease ecology at the beginning of the week, the underserved group improved their understanding of disease ecology more from the camp activities than the comparison group.

A strong component of the model for both groups was relevance. For the undergraduate researchers, the relevance was threefold 1) creating activities connected to their majors and/or research, 2) enhancing their skills and interest in science communication (post surveys) and 3) their own interest in disease ecology. For the youth, the connection to their culture was especially important for Indigenous youth but intriguing to most of the youth (Bruchac, 2016; Windchief and San Pedro, 2019). Learning more about diseases in their state was relevant for all the youth since many knew someone affected by them or had at least heard of them. The emphasis on what they can do to stay healthy while interacting with their own environments made the science relevant to their lives and the lives of their families and communities.

Since health and disease are inherently interesting to people because they want to be healthy, the core ideas of how the environment, living things, and science and culture interact to affect health captured and held youths’ interest. By being place-based and integrating Native culture, the underserved group was just as engaged and had equivalent or better outcomes (Avery, 2013; McInerny, et al., 2011). They started to put together how their decisions affect their health and how to stay safe in an environment that may contain pathogens. This approach has been shown to be particularly effective for strengthening youths’ connections to their communities (Bouchard and Wike, 2022), a core value of Indigenous communities (Sobel, 2004).

The project team learned several lessons throughout this work. Undergraduates’ lack of experience with creating activities and with teaching in general made it challenging for them to facilitate the activities. Pairing the undergraduates with educators early in the process of designing the activities could address this challenge. Additionally, having experienced educators teach the camp, assisted by undergraduates could provide a better experience for both the undergraduates and the campers. Another lesson learned was that having youth create original concept maps of disease ecology was a struggle for those who did not feel comfortable writing. Instead of creating maps from scratch, using the visual model as a touchstone they can add to may enhance the development of their understanding. Youth were shown the model briefly on the first day, but returning to the core ideas in the visual model (Figure 1) each session may have provided more context and helped build concepts throughout the week. Anchoring the activities in the core concepts would would also aid educators in replicating the activities in the future.

Limitations to this study include some missing data from the undergraduates at the end of the academic year, the small number of youth in the camps, some missing data from the comparison camp, and a lack of different ways for youth to reflect on what they were learning. Perhaps verbal or video products for an authentic audience would be more effective in focusing youth on learning the concepts and communicating their understanding to their families and communities.

An implication of this study is that engaging undergraduates in science outreach and communication will not only build their skills, but also play a meaningful role in their own education. Undergraduates in various science fields appreciated the opportunity to learn about and do outreach with youth in order to develop young people’s knowledge and enthusiasm for science, improve their own understanding of science, and to promote their own scientific research (Varner, 2014). Scientists are becoming increasingly aware of the need to communicate with the public, not just their peers, so undergraduate experience in a low-stakes environment can build confidence and skill (Stony Brook University, 2009). In this model, the undergraduates focused on what they thought would engage middle school youth in the design of their activities and the resulting activities were indeed engaging, reinforcing their confidence in doing outreach (Volbrecht, 2019). Many reported that the collaboration with other students was a rewarding opportunity they had not often had.

A second implication of this study is that having a topic like disease ecology that is engaging for middle school youth results in increased interest and confidence in STEM. In this project, they wanted to know more about how to protect and enhance their health and the health of their families and community. Understanding microbes, diseases, and transmission in their own environments through a place-based approach was enough of a hook that they wanted to learn more. To retain and share what they have learned with others, they probably need more opportunities to present, perhaps through short videos or posters, extending their learning through communicating with authentic audiences in their communities.

A third implication is that activities that are problem-based, place-based, and involve teams are effective with diverse youth, regardless of race or socioeconomic status. They were all interested and learned from the activities.

We provide evidence that involving undergraduates benefitted them as aspiring scientists who now value science communication and outreach and feel more confident and skilled to do it in the future. A follow up study is needed on the extent to which they pursue outreach opportunities in the future. Middle school youth also benefitted from the approach. Further study on the effects of this model on underserved middle school youth over time is needed. Do they use what they learned? Do they share it with others? Do they pursue more STEM opportunities? The next phase of the project involves training formal and informal educators to use the activities with their audiences with the goal of achieving similar levels of engagement and outcomes with youth. We need to better understand how the nature and extent of their use of the activities affects the outcomes.

## Figures and Tables

**Table 1.** Finalized Undergraduate Activities for Camp.

Time	Topic	Activities
Day 1	Introduction to disease ecology and microbes	Youth hypothesize about which objects around them might be harboring the most microbes, then swab samples inside and outside the building, placing them onto agar plates for analysis on Friday.
Day 2	Isolating and identifying microbes on or around you	Youth identify microbes in their region associated with diseases like Rocky Mountain Spotted Fever, Coronavirus, Blue Tongue, and E.coli. Working in teams, they play a game using keys and locked boxes (puzzles) to discover if their illness was caused by bacteria or a virus, if it was contagious, and if so how, and what the treatment would be. They present their findings to the other groups. In a second activity, they review case studies with photos, data, and a list of symptoms to identify diseases found locally. For example, “After hiking in the Bitterroot Valley, a 30-year-old male comes into the emergency room with high fever and a spotted rash which started around his hands and feet. They think he may have been bitten by a tick.”
Day 3	Exploring water systems in our state and sources of pollution and collecting samples	Youth collect samples from water sources on campus and learn about watersheds in the state, waterborne microbes, sources of pollution, and pH and dissolved oxygen levels in water. Then they analyze their own sample and hypothesize about the reasons for its condition. They also learn about the perspectives of indigenous water advocates.
Day 4	Nutrition and Native science	Youth hear stories about how important food is to health, by studying nutritional labels (US and EU), and understanding taste through sampling different foods. Youth explore the food and culture of their families and communities.
Day 5	Microplastics, water filtration, algal blooms	Youth look at their agar plates and predictions from Monday’s swabs and at the microbes and microplastics in their water samples from Wednesday. Then they look at types and sources of microplastics and learn about algal blooms in local water and how they impact pH and dissolved oxygen and plan how to improve their own water quality.

**Table 2.** Effects on Undergraduates: Self-Reported on Retrospective Question at End of Year (1–10).

	Mean Response		
	Before	After	Gain
Interest in science communication and outreach	7.0	8.8	1.8
See how science outreach could be used to help my community	5.3	8.7	3.4
Skills in science communication and outreach	4.9	7.7	2.8
Confidence in my ability to do science communication and outreach	4.5	7.8	3.3

**Table 3.** Beginning to End-of-Year Disease Ecology Concept Map Scores for Undergraduates (Novak and Gowin, 1984).

Undergraduates	Pre	Post	% increase
Average	85.2	141.5	66%

**Table 4.** Youth STEM Identity Before and After Camp by Group. Group sizes: Underserved N=21; Comparison N=13; B=Before; A=After; SD=standard deviation; SEM=standard error of mean.

Underserved group				Comparison group			
Before	After	SD	SEM	Before	After	SD	SEM
3.3	5.4*	1.63	0.225	3.6	5.1*	1.47	0.176

* p<0.05

**Table 5.** Youth Understanding of Disease Ecology by Group. Table 5 shows the results of a retrospective question asked of each group **at the end of their camp**. The question read: **Rate your understanding before and after this camp of each of the ideas below on a scale of 1–10**. Group sizes: Underserved N=21; Comparison N=13; SD=standard deviation; SEM=standard error of mean.

Understanding of:	Underserved group				Comparison group			
	Before	After	SD	SEM	Before	After	SD	SEM
Disease ecology	4.4	8.0*	2.8	0.112	4.5	7.8*	2.3	0.136
Native culture	6.2	7.2*	2.3	0.136	5.5	6.2*	2.3	0.201
Traditional knowledge about food	5.7	7.2*	2.8	0.132	5.1	6.2*	2.2	0.143
How to be healthy where we live	5.3	8.2*	2.8	0.141	7.4	9.1*	1.8	0.129
How science can help my community	5.9	7.8*	2.7	0.146	6.7	7.9*	2.4	0.128
**Averages**	**5.5**	**7.7**			**5.8**	**7.4**		

* p<0.05

**Table 6.** Youth Pre/Post Disease Ecology Concept Map Scores.

Underserved N=23			Comparison N=16		
Pre	Post	Score Change	Pre	Post	Score Change
9.8	78.4	+68.6	8.5	45.3	+36.8

## ABBREVIATIONS

INBRE: IDeA Networks of Biomedical Research Excellence
NESSP: Northwest Earth and Space and Sciences Pathways
NGSS: Next Generation Science Standards
SEPA: Science Education Partnership Award
